# Supplementary material for: Evolutionary history of zoogeographical regions surrounding the Tibetan Plateau
Source: Commun Biol. 2020 Jul 31;3:415. doi: 10.1038/s42003-020-01154-2 (PMC7395132; doi:10.1038/s42003-020-01154-2)
Supplement: Supplementary file 1 — Supplementary Information [file 42003_2020_1154_MOESM1_ESM.pdf]

Supplementary Information

Evolutionary history of zoogeographical regions surrounding the Tibetan Plateau

This PDF file includes:

Supplementary Figures 1–5

Supplementary Tables 1–2

Supplementary Methods

Supplementary References

Supplementary Figures

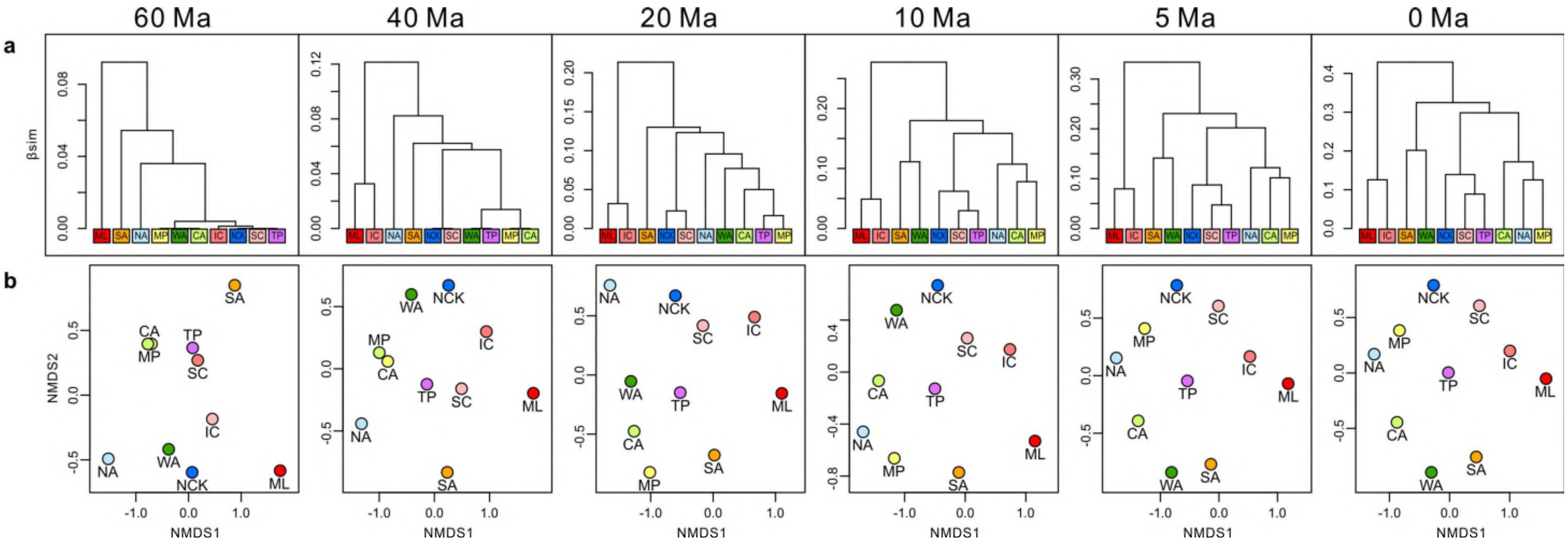

Supplementary Figure 1 Temporal changes in zoogeographical regions surrounding the Tibetan Plateau at successive phylogenetic depths during the Cenozoic for the whole-region species list of all terrestrial vertebrates. a. Dendrograms plotted by the unweighted pair-group method using arithmetic average clustering. **b**. Non- metric multidimensional scaling ordinations based on the $p\beta_{sim}$ matrices of whole-region species list of all terrestrial vertebrates

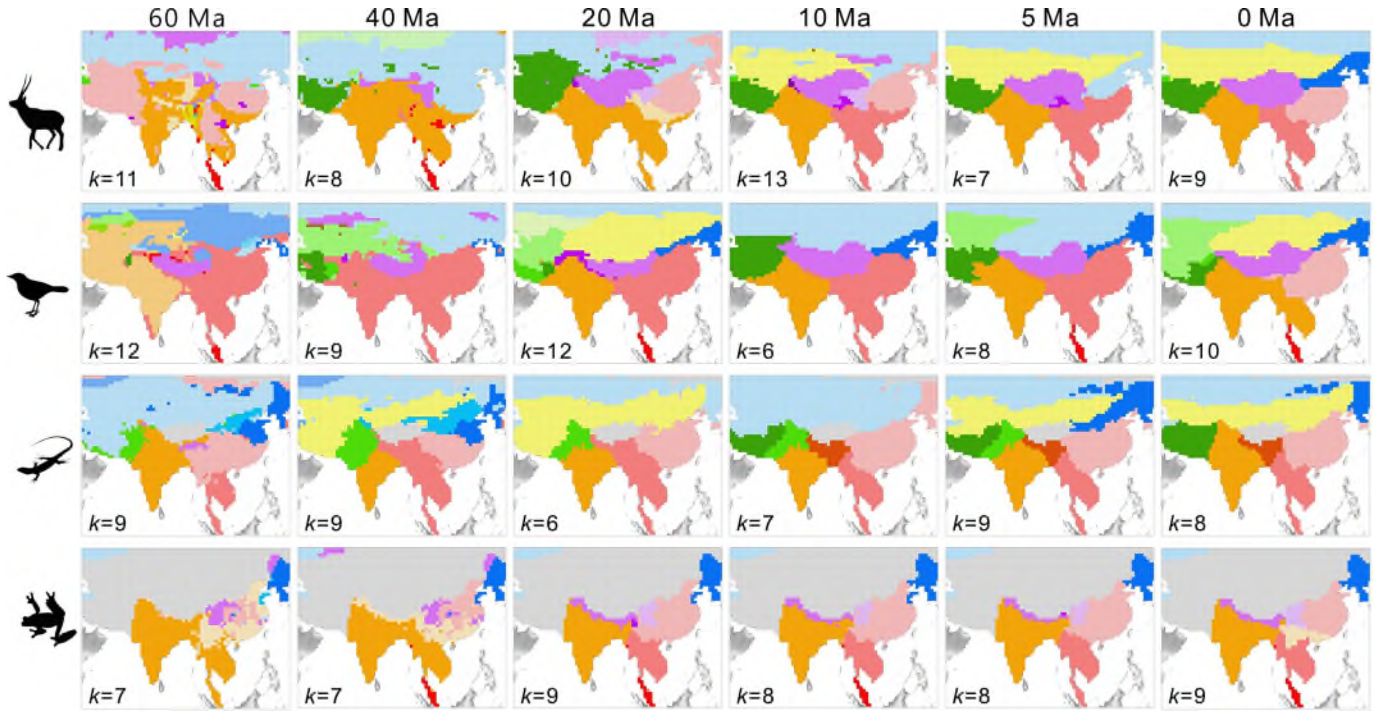

Supplementary Figure 2 Temporal changes in the zoogeographical regions surrounding the Tibetan Plateau at successive phylogenetic depths during the Cenozoic for four vertebrate classes. The zoogeographical regions were generated based on $p\beta_{sim}$ dissimilarity between pairs of grid-based communities for individual vertebrate classes (mammals, birds, reptiles, and amphibians). The optimal cluster number for each time interval was based on mean silhouette width and explained dissimilarity (Supplementary Data 3). In the amphibian and reptile maps, grid cells with species richness < 5 are in grey.

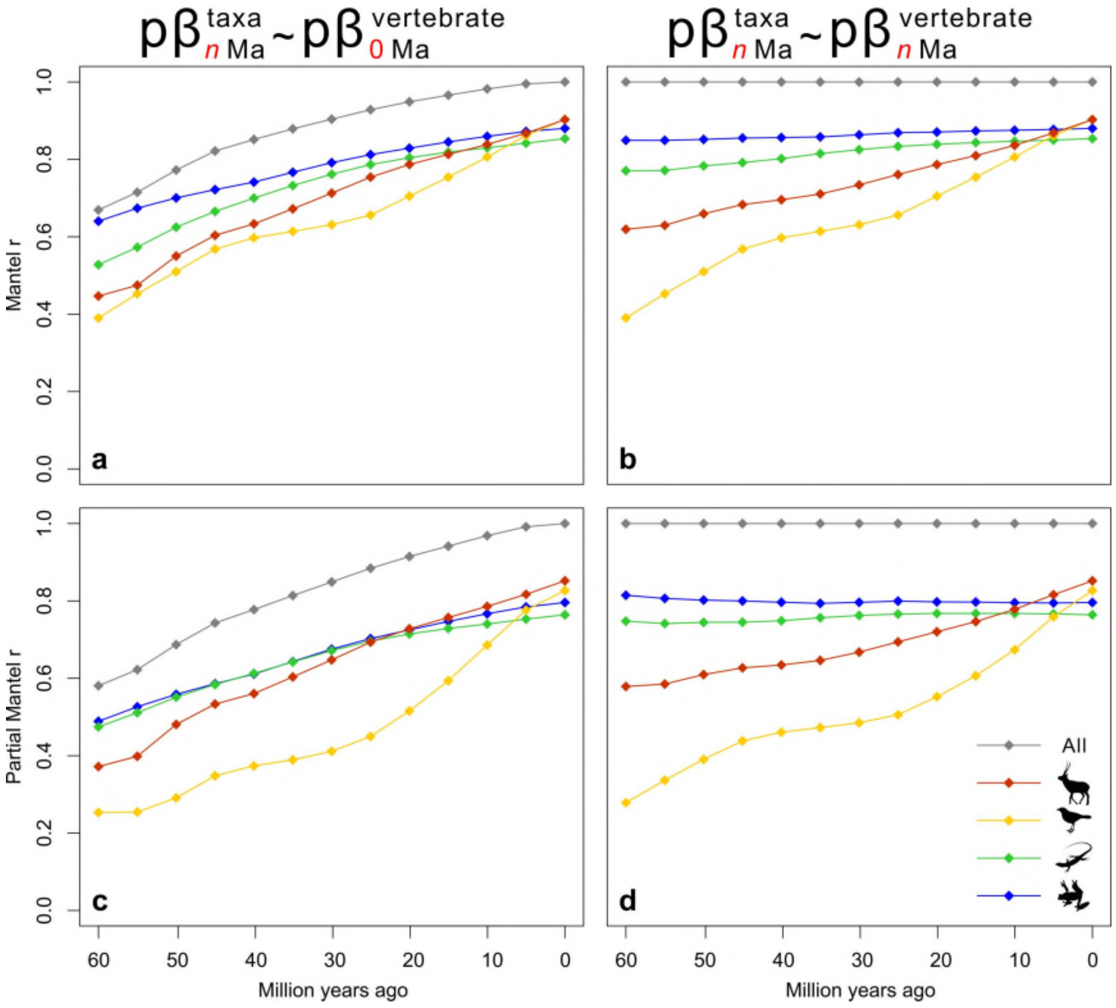

Supplementary Figure 3 Mantel and partial Mantel correlation tests between the pβ_{sim} matrices of each vertebrate class and all terrestrial vertebrates. a. Mantel correlation tests between each vertebrate class in a given time interval and all terrestrial vertebrates at present. **b**. Mantel correlation tests between each vertebrate class and all terrestrial vertebrates in a given time interval. **c** and **d**. Partial Mantel tests with a matrix of pairwise Euclidean distances as a covariate. All tests were highly significant ($P < 0.001$)

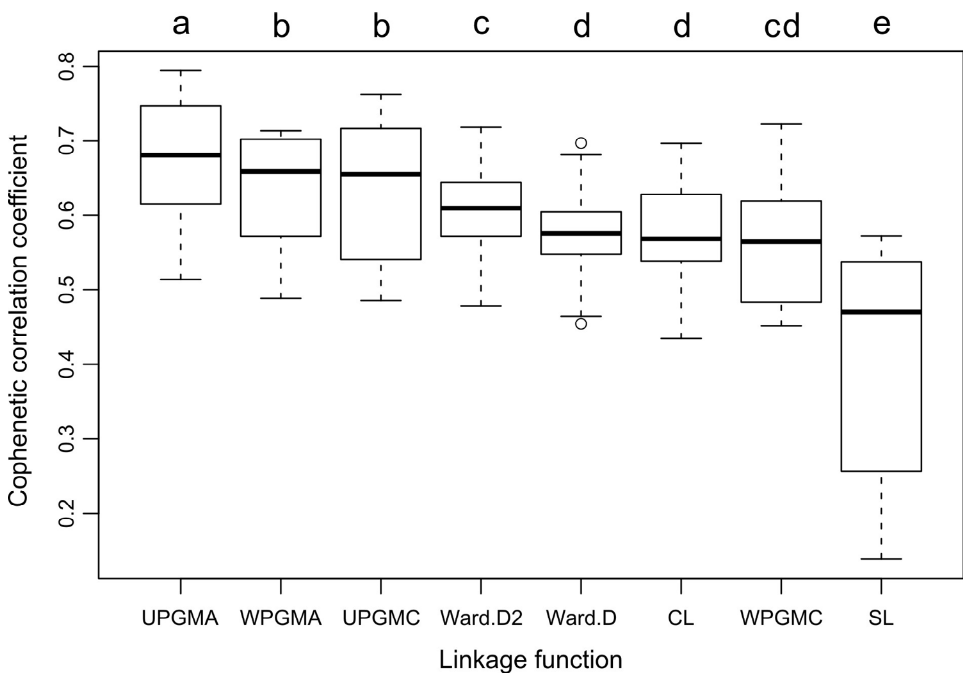

Supplementary Figure 4 Boxplots showing co-phenetic correlation coefficients of eight different clustering algorithms across 13 phylogenetic depths from 60 to 0 Ma, at intervals of 5 Myr time bins. Boxes show the median and 25th and 75th percentiles. The letters indicate significant differences among eight clustering algorithms (Wilcoxon Signed Rank Tests, $P < 0.05$).

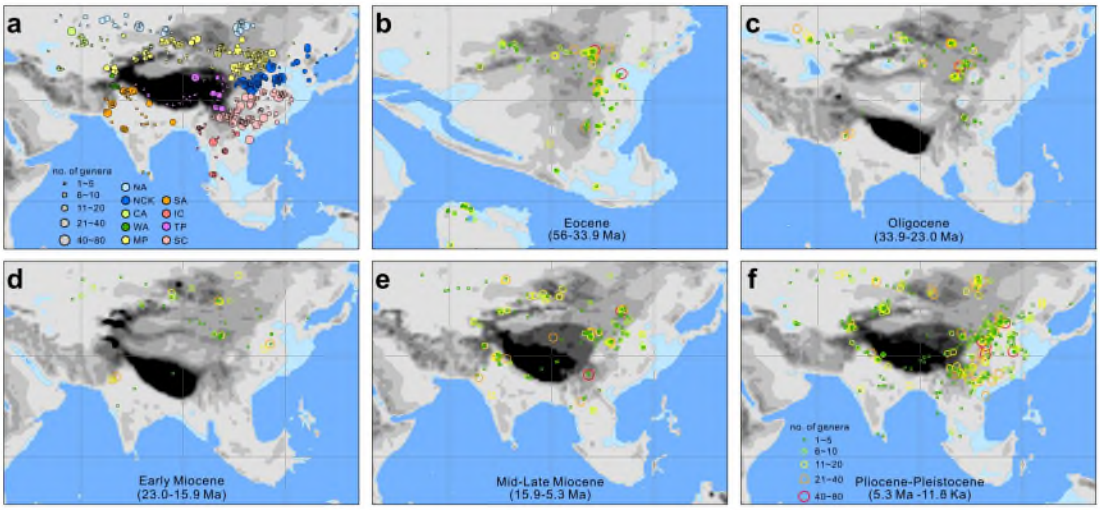

Supplementary Figure 5 Spatial and temporal distribution of fossil assemblages during the Cenozoic. a. Fossil assemblages with different colors indicating their assignments within different zoogeographical region. **b–f**. Distributions of fossil assemblages during different time periods. The size of the symbol is proportional to the number of mammal genera in each assemblage.

Supplementary Tables

Supplementary Table 1 Mantel correlations (Spearman's rho) among pairs of time slices based on β_{sim} matrices at the genus level (below the diagonal). *P* values are listed above the diagonal with $P < 0.05$ in bold. Plio-Pleis: Pliocene–Pleistocene.

Epoch	Present	Plio-Pleis	Mid-Late Miocene	Early Miocene	Oligocene	Eocene
Present	—	**0.019**	0.053	0.229	0.079	**0.022**
Plio-Pleis	**0.3645**	—	**0.020**	0.987	0.278	**0.048**
Mid-Late Miocene	0.3490	**0.5131**	—	**0.047**	0.145	0.211
Early Miocene	0.1789	-0.5619	**0.5139**	—	0.357	0.620
Oligocene	0.3048	0.1351	0.2894	0.1423	—	0.393
Eocene	**0.4606**	**0.4796**	0.2715	-0.0995	0.0898	—

Supplementary Table 2 Proportion of observed genera of the number of those expected by the Chao2 estimator for each time interval. The number of fossil localities within the zoogeographical regions is in parenthesis. '—', indicates faunas not sampled or with fewer than five genera. Plio-Pleis: Pliocene–Pleistocene.

Region	Plio-Pleis	Mid-Late Miocene	Early Miocene	Oligocene	Eocene
Central Asia	63.7(10)	—	28.6(2)	54.1(17)	33.1(2)
Indochina	53.6(14)	35.9(7)	—	100(1)	7.5(12)
Mongolian Plateau	62.4(33)	67.3(36)	56.9(14)	81.3(49)	73(54)
North Asia	76.6(32)	26.8(6)	61.2(4)	100(1)	—
North China & Korea	54.1(64)	55.3(21)	39(4)	38.5(5)	44.3(30)
South Asia	68.8(18)	68.8(12)	7.7(4)	7.3(4)	26.1(7)
South China	68.5(67)	11.7(7)	100(1)	14.5(10)	48.2(35)
Tibetan Plateau	32.3(23)	6.5(8)	21.4(3)	100(1)	50(2)
West Asia	29.2(4)	11.5(3)	—	—	—
Mean±SD (n)	56.6±15.4(265)	41.1±27.5(103)	45±28.5(32)	62±36.3(88)	40.3±19.2(142)

Supplementary Method

Dissimilarity index

We used Simpson's dissimilarity index (β_{sim}) and Simpson's phylogenetic dissimilarity index ($p\beta_{sim}$) to generate pairwise dissimilarity for each pair of communities (1). These two dissimilarity indices represent species (or phylogenetic) turnover independent of the total species richness (or branch length) difference between two communities (2).

The β_{sim} index calculates the pairwise species dissimilarity distance between two communities as follows:

$$\beta_{\text{sim}} = 1 - \frac{a}{\min(b, c) + a}$$

where a is the number of shared species and b and c are the number of species unique to each grid cell. β_{sim} ranges from 0 to 1, with smaller values indicating lower dissimilarity.

The $p\beta_{sim}$ index calculates the pairwise phylogenetic dissimilarity distance between two grid cells as follows:

$$p\beta_{\text{sim}} = \frac{\min(\text{PD}_{Total} - \text{PD}_k,\ \text{PD}_{Total} - \text{PD}_j)}{\text{PD}_k + \text{PD}_j - \text{PD}_{Total} + \min(\text{PD}_{Total} - \text{PD}_k,\ \text{PD}_{Total} - \text{PD}_j)}$$

where PD_j and PD_k are the total branch lengths of communities *j* and *k*, respectively. PD_{Total} is the total branch length of a phylogenetic tree containing all species present in both communities *j* and *k*. $p\beta_{sim}$ ranges from 0 (species are identical and share the same branch lengths) to 1 (species share no phylogenetic branches).

Determining the optimal number of clusters

We implemented the approach of Holt et al. (3) to select suitable dendrogram cutoff points based on the explained dissimilarity and mean silhouette width. The explained dissimilarity is the ratio of the sums of mean dissimilarity within different clusters to the total matrix dissimilarity. This value tends to 1 when all areas are considered as independent groups. The silhouette width assesses the clustering solutions and ranges between -1 and +1. A negative silhouette width indicates that most cells are probably located in an incorrect cluster (4). We only considered the alternate cluster numbers in the range of 2–15, as many regions were difficult to recognize and explain.

Cross-taxon analyses

For four individual vertebrate classes, we generated separate $p\beta_{sim}$ matrices between gridded species assemblages at different phylogenetic depths. Similarly, we ran UPGMA clustering and NMDS ordinations to investigate the changes in assignments and topologies of clustering dendrograms over a phylogenetic timescale. Besides, we used Mantel tests to calculate the correlation coefficients of the phylogenetic dissimilarity matrices ($p\beta_{sim}$) between each vertebrate class and all terrestrial vertebrates in different time slices. To account for the spatial autocorrelation (i.e. geographical distance), we repeated these analyses using the partial Mantel tests. Statistical significance was calculated with a Mantel Carlo permutation test using 999 permutations.

Analyses based on whole-region mammal list

Because we merged the fossil records to coarse zoogeographical regions to infer the changes in fossil assemblages, and to maximize the comparison of fossil data and molecular phylogeny, we calculated the phylogenetic dissimilarities between zoogeographical regions based on the species lists for the whole region. In this analysis, we obtained the whole-region species lists by combining grid cells belonging to the zoogeographical regions identified by the present-day phylogenetic beta diversity. Then, we calculated $p\beta_{sim}$ between each pair of zoogeographical regions at different phylogenetic depths and performed UPGMA clustering and NMDS ordinations. Finally, we assessed the differences in the UPGMA dendrograms and NMDS ordinations based on gridded assemblages versus whole-region species lists.

Supplementary References

1. Baselga, A. Partitioning the turnover and nestedness components of beta diversity. *Global Ecol. Biogeogr.* **19**, 134-143 (2010).
2. Leprieur, F. & Oikonomou, A. The need for richness - independent measures of turnover when delineating biogeographical regions. *J. Biogeogr.* **41**, 417-420 (2014).
3. Holt, B. G. *et al.* An update of Wallace's zoogeographic regions of the world. *Science* **339**, 74-78 (2013).
4. Dapporto, L. *et al.* A new procedure for extrapolating turnover regionalization at mid-small spatial scales, tested on British butterflies. *Methods Ecol. Evol.* **6**,

1287-1297 (2015).
