# Supplementary material for: Evolutionary history of zoogeographical regions surrounding the Tibetan Plateau
Source: Commun Biol. 2020 Jul 31;3:415. doi: 10.1038/s42003-020-01154-2 (PMC7395132; doi:10.1038/s42003-020-01154-2)
Supplement: Supplementary file 5 — Description of Additional Supplementary Files [file 42003_2020_1154_MOESM5_ESM.pdf]

Description of Additional Supplementary Files

Supplementary Data 1 Checklist and distribution of extant terrestrial vertebrates

Supplementary Data 2 Checklist and distribution of extinct mammal genera

Supplementary Data 3 Optimal cluster number for each time interval
