# Supplementary material for: Evolutionary history of zoogeographical regions surrounding the Tibetan Plateau
Source: Commun Biol. 2020 Jul 31;3:415. doi: 10.1038/s42003-020-01154-2 (PMC7395132; doi:10.1038/s42003-020-01154-2)
Supplement: Supplementary file 6 — Reporting Summary [file 42003_2020_1154_MOESM6_ESM.pdf]

natureresearch

Corresponding author(s): Haisheng, Jiang

Last updated by author(s): Jun 22, 2020

Reporting Summary

Nature Research wishes to improve the reproducibility of the work that we publish. This form provides structure for consistency and transparency in reporting. For further information on Nature Research policies, see Authors & Referees and the Editorial Policy Checklist.

Statistics

For all statistical analyses, confirm that the following items are present in the figure legend, table legend, main text, or Methods section.

n/a	Confirmed	
☐	☒	The exact sample size (*n*) for each experimental group/condition, given as a discrete number and unit of measurement
☐	☒	A statement on whether measurements were taken from distinct samples or whether the same sample was measured repeatedly
☐	☒	The statistical test(s) used AND whether they are one- or two-sided<br>*Only common tests should be described solely by name; describe more complex techniques in the Methods section.*
☒	☐	A description of all covariates tested
☒	☐	A description of any assumptions or corrections, such as tests of normality and adjustment for multiple comparisons
☐	☒	A full description of the statistical parameters including central tendency (e.g. means) or other basic estimates (e.g. regression coefficient) AND variation (e.g. standard deviation) or associated estimates of uncertainty (e.g. confidence intervals)
☐	☒	For null hypothesis testing, the test statistic (e.g. *F*, *t*, *r*) with confidence intervals, effect sizes, degrees of freedom and *P* value noted<br>*Give P values as exact values whenever suitable.*
☒	☐	For Bayesian analysis, information on the choice of priors and Markov chain Monte Carlo settings
☒	☐	For hierarchical and complex designs, identification of the appropriate level for tests and full reporting of outcomes
☒	☐	Estimates of effect sizes (e.g. Cohen's *d*, Pearson's *r*), indicating how they were calculated

Our web collection on statistics for biologists contains articles on many of the points above.

Software and code

Policy information about availability of computer code

Data collection	No software was used to collect data
Data analysis	All analyses were conducted in the programming environment R version 3.6.0. The most important R packages were 'betapart', 'recluster', 'vegan', and 'picante'. All raw data and custom R codes are available from the Dryad Digital Repository (https://doi.org/10.5061/dryad.5x69p8d10).

For manuscripts utilizing custom algorithms or software that are central to the research but not yet described in published literature, software must be made available to editors/reviewers. We strongly encourage code deposition in a community repository (e.g. GitHub). See the Nature Research guidelines for submitting code & software for further information.

Data

Policy information about availability of data

All manuscripts must include a data availability statement. This statement should provide the following information, where applicable:

- Accession codes, unique identifiers, or web links for publicly available datasets
- A list of figures that have associated raw data
- A description of any restrictions on data availability

The species geographical ranges were based on the IUCN Red List database (http://www.iucnredlist.org), Birdlife International and NatureServe (http://www.birdlife.org), Global Biodiversity Information Facility (GBIF; http://www.gbif.org), and Roll et al. (2017; https://doi.org/10.5061/dryad.83s7k). The phylogenies for four vertebrate classes were available from the VertLife dataset online (http://vertlife.org/phylosubsets). The fossil data was compiled from the Institute of Vertebrate Paleontology and Paleoanthropology, Beijing (http://www.ivpp.ac.cn/); the Paleobiology Database (https://www.paleobiodb.org/); the New and Old Worlds database (https://www.helsinki.fi/science/now/); and the Fossilworks database (https://fossilworks.org/). The data supporting the findings of this study are available from Dryad Digital Repository (https://doi.org/10.5061/dryad.5x69p8d10).

Field-specific reporting

Please select the one below that is the best fit for your research. If you are not sure, read the appropriate sections before making your selection.

☐ Life sciences ☐ Behavioural & social sciences ☒ Ecological, evolutionary & environmental sciences

For a reference copy of the document with all sections, see nature.com/documents/nr-reporting-summary-flat.pdf

Ecological, evolutionary & environmental sciences study design

All studies must disclose on these points even when the disclosure is negative.

Study description	To reconstruct the evolutionary history of the zoogeographical regions surrounding the TP, we used 4,966 extant terrestrial vertebrates and 1,278 extinct mammal genera to quantify the temporal changes in beta dissimilarities. We calculated phylogenetic beta dissimilarities at different phylogenetic depths using species distributions and dated phylogenies extant terrestrial vertebrates, and calculated beta dissimilarity over geological time using extinct mammal genera.
Research sample	For extant species, we sampled all terrestrial vertebrates on the Tibetan Plateau and surrounding regions based on the distribution data. We excluded introduced, marine, domestic species, and species that cannot match the phylogenetic tips. For fossil data, we sampled all mammal fossils during the Cenozoic era, and excluded taxa unidentifiable at the genus level.
Sampling strategy	does not apply
Data collection	Species range polygons are available upon request from the IUCN Red List website (http://www.iucnredlist.org) for mammals and amphibians, Birdlife International and NatureServe (http://www.birdlife.org) for birds, and Roll et al. (2017) for reptiles. The phylogenies of terrestrial vertebrates included in the study are all publicly available online (http://vertlife.org/phylosubsets; Upham et al., 2019; Jetz et al., 2014; Jetz & Pyron, 2018; Tonini et al., 2016). The fossil records are available from online databases, including the Institute of Vertebrate Paleontology and Paleoanthropology, Beijing (http://www.ivpp.ac.cn/); the Paleobiology Database (PBDB; https://www.paleobiodb.org/); the New and Old Worlds database (https://www.helsinki.fi/science/now/); and the Fossilworks database (https://fossilworks.org/).
Timing and spatial scale	The geographical ranges of extant species were downloaded from public databases in July, 2017 for mammals, birds, amphibians, and in November, 2019 for reptiles. The fossil records of mammals were compiled in August, 2019. The spatial scale is the Tibetan Plateau and its surrounding regions.
Data exclusions	For species, we excluded introduced, marine and domestic species. Besides, we combined the distributional and phylogenetic data, and excluded species that did not match. For grid cells, we removed grid cells with land area < 50% and species richness < 5.
Reproducibility	All analyses are performed within the R statistical environment, and the code and the data for all analyses is deposited in the Dryad Digital Repository.
Randomization	does not apply
Blinding	does not apply

Did the study involve field work? ☐ Yes ☒ No

Reporting for specific materials, systems and methods

We require information from authors about some types of materials, experimental systems and methods used in many studies. Here, indicate whether each material, system or method listed is relevant to your study. If you are not sure if a list item applies to your research, read the appropriate section before selecting a response.

Materials & experimental systems

n/a	Involved in the study	
☒	☐	Antibodies
☒	☐	Eukaryotic cell lines
☐	☒	Palaeontology
☒	☐	Animals and other organisms
☒	☐	Human research participants
☒	☐	Clinical data

Methods

n/a	Involved in the study	
☒	☐	ChIP-seq
☒	☐	Flow cytometry
☒	☐	MRI-based neuroimaging

Palaeontology

Specimen provenance	The fossil records are available from online databases, including the Institute of Vertebrate Paleontology and Paleoanthropology,

Specimen provenance	Beijing (http://www.ivpp.ac.cn/); the Paleobiology Database (PBDB; https://www.paleobiodb.org/); the New and Old Worlds database (https://www.helsinki.fi/science/now/); and the Fossilworks database (https://fossilworks.org/).
Specimen deposition	All the fossil collections are available from public databases.
Dating methods	does not apply

☒ Tick this box to confirm that the raw and calibrated dates are available in the paper or in Supplementary Information.
